# The impact of the temporary suspension of national cancer screening programmes due to the COVID-19 epidemic on the diagnosis of breast and colorectal cancer in the Netherlands

**DOI:** 10.1186/s13045-020-00984-1

**Published:** 2020-11-04

**Authors:** Avinash G. Dinmohamed, Matteo Cellamare, Otto Visser, Linda de Munck, Marloes A. G. Elferink, Pieter J. Westenend, Jelle Wesseling, Mireille J. M. Broeders, Ernst J. Kuipers, Matthias A. W. Merkx, Iris D. Nagtegaal, Sabine Siesling

**Affiliations:** 1 Department of Research and Development, Netherlands Comprehensive Cancer Organisation (IKNL), Godebaldkwartier 419, 3511 DT Utrecht, The Netherlands; 2 Department of Public Health, Erasmus MC, University Medical Center Rotterdam, Rotterdam, The Netherlands; 3 Department of Hematology, Cancer Center Amsterdam, Amsterdam UMC, Amsterdam, The Netherlands; 4 Department of Registration, Netherlands Comprehensive Cancer Organisation (IKNL), Utrecht, The Netherlands; 5 Department of Pathology, Albert Schweitzer Hospital, Dordrecht, The Netherlands; 6 Laboratory for Pathology Dordrecht, Dordrecht, The Netherlands; 7 Divisions of Diagnostic Oncology and Molecular Pathology, The Netherlands Cancer Institute, Amsterdam, The Netherlands; 8 Department of Pathology, Leiden University Medical Center, Leiden, The Netherlands; 9 Radboud Institute for Health Sciences, Radboud University Medical Center, Nijmegen, The Netherlands; 10 Dutch Expert Centre for Screening, Nijmegen, The Netherlands; 11 Erasmus MC, University Medical Center Rotterdam, Rotterdam, The Netherlands; 12 Netherlands Comprehensive Cancer Organisation (IKNL), Utrecht, The Netherlands; 13 Department of Oral and Maxillofacial Surgery, Radboud University Medical Center, Nijmegen, The Netherlands; 14 PALGA Foundation, Houten, The Netherlands; 15 Department of Pathology, Radboud University Medical Center, Nijmegen, The Netherlands; 16 Department of Health Technology and Services Research, Technical Medical Centre, University of Twente, Enschede, The Netherlands

**Keywords:** COVID-19, Cancer, Incidence, Epidemiology, Registry, Population-based, Screening

## Abstract

Oncological care was largely derailed due to the reprioritisation of health care services to handle the initial surge of COVID-19 patients adequately. Cancer screening programmes were no exception in this reprioritisation. They were temporarily halted in the Netherlands (1) to alleviate the pressure on health care services overwhelmed by the upsurge of COVID-19 patients, (2) to reallocate staff and personal protective equipment to support critical COVID-19 care, and (3) to mitigate the spread of COVID-19. Utilising data from the Netherlands Cancer Registry on provisional cancer diagnoses between 6 January 2020 and 4 October 2020, we assessed the impact of the temporary halt of national population screening programmes on the diagnosis of breast and colorectal cancer in the Netherlands. A dynamic harmonic regression model with ARIMA error components was applied to assess the observed versus expected number of cancer diagnoses per calendar week. Fewer diagnoses of breast and colorectal cancer were objectified amid the early stages of the initial COVID-19 outbreak in the Netherlands. This effect was most pronounced among the age groups eligible for cancer screening programmes, especially in breast cancer (age group 50–74 years). Encouragingly enough, the observed number of diagnoses ultimately reached and virtually remained at the level of the expected values. This finding, which emerged earlier in age groups not invited for cancer screening programmes, comes on account of the decreased demand for critical COVID-19 care since early April 2020, which, in turn, paved the way forward to resume screening programmes and a broad range of non-critical health care services, albeit with limited operating and workforce capacity. Collectively, transient changes in health-seeking behaviour, referral practices, and cancer screening programmes amid the early stages of the initial COVID-19 epidemic in the Netherlands conjointly acted as an accelerant for fewer breast and colorectal cancer diagnoses in age groups eligible for cancer screening programmes. Forthcoming research is warranted to assess whether the decreased diagnostic scrutiny of cancer during the COVID-19 pandemic resulted in stage migration and altered clinical management, as well as poorer outcomes.

**To the Editor,**

The chaos wreaked by COVID-19 catalysed a notable decrease in cancer diagnoses in the Netherlands compared with the period preceding the COVID-19 outbreak [1]. At the time when these findings were published, provisional data from the Netherlands Cancer Registry (NCR) on cancer diagnoses were available up to 12 April 2020 [1]. Therefore, the impact of the temporary halt of national population screening programmes for breast and colorectal cancer—which were halted as of 16 March 2020—could not yet be disentangled with the comparatively short observation period [1]. These programmes were halted to ease the burden on health-care services overwhelmed by the surge of COVID-19 patients, to reallocate personal protective equipment (PPE) to health care staff tackling COVID-19, and to mitigate the spread of COVID-19.

The demand for critical COVID-19 care steadily decreased in the Netherlands since early April 2020. Consequently, hospital capacity for the diagnostic work-up of suspected cancer cases gradually re-established and PPE became increasingly available for a broad range of health care workers (e.g. radiographers and colonoscopists). Also, cancer screening units and waiting rooms were reorganised to minimise contracting COVID-19 in such environments. Therefore, invitations to screening programmes for colorectal and breast cancer gradually recommenced—albeit with limited operating and workforce capacity—as of mid-May 2020 and mid-June 2020, respectively.

With more recent data available on cancer diagnoses up to 4 October 2020, we assessed the impact of the temporarily suspended national screening programmes on the initial pathological notification of ductal carcinoma in situ (DCIS) and invasive breast cancer—hereafter collectively designated as breast cancer—and colorectal cancer in the Netherlands.

We selected patients diagnosed between 6 January 2020 and 4 October 2020 from the NCR that relies on pathological cancer notifications via the Nationwide Histopathology and Cytopathology Data Network and Archive. Of note, colorectal adenomas are not ascertained in the NCR. The expected number of newly diagnosed malignancies per calendar week during the study period was predicted using a dynamic harmonic regression model with ARIMA error components based on the observed weekly trends in cancer diagnoses in the period 2010–2019. The Additional file 1 provides methodological details.

Breast cancer diagnoses among women aged < 50 or > 74 years (i.e. those not invited for biennial mammography screening) became significantly lower—as compared to the expected number of diagnoses—as of mid-March (Fig. 1a), owing to changes in health-seeking behaviour and referral practices amid the early stages of the COVID-19 epidemic [1]. Encouragingly enough—as of early May—the observed number of diagnoses in these age groups was reached and virtually remained at the level of the expected values. The number of breast cancer diagnoses among women aged 50–74 years (i.e. those invited for biennial mammography screening) showed a very steep decline as of early April—that is, 2 weeks after the suspension of breast cancer screening (Fig. 1b). Thereafter, the number of diagnoses remained lower than the expected number of diagnoses until mid–late June. The trends described herein were commensurate between invasive breast cancer and DCIS (Additional file 1).

**Fig. 1:**
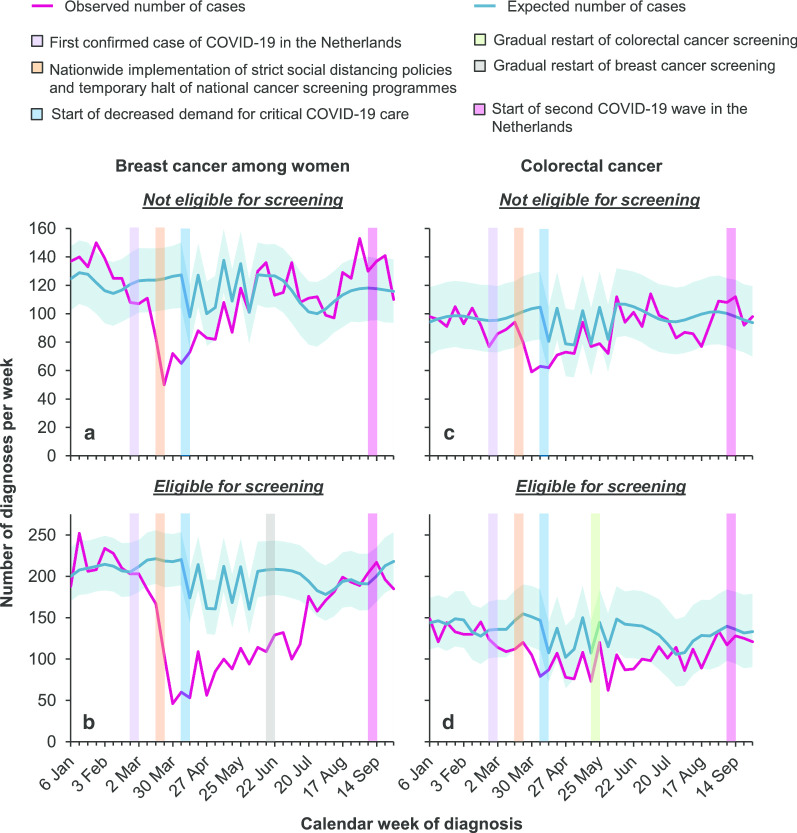
The weekly number of breast and colorectal cancer diagnoses in the Netherlands between 6 January 2020 and 4 October 2020. The difference between the observed (pink line) and expected number of cancer diagnoses (blue line) is considered statistically significant when the observed number of cancer diagnoses does not fall within the range of the 95% confidence intervals of the expected number of cancer diagnoses (blue shaded area). **a**, **b** The observed and expected number of breast cancer diagnoses among women age < 50 or > 74 years (i.e. those not invited for biennial mammography screening) and women aged 50–74 years (i.e. those invited for biennial mammography screening), respectively. **c**, **d** The observed and expected number of colorectal cancer diagnoses among individuals age < 55 or > 75 years (i.e. those not invited for biennial faecal immunochemical testing) and individuals aged 55–75 years (i.e. those invited for biennial faecal immunochemical testing), respectively. The current statistics do not yet include cases diagnosed in one of the 74 hospitals in the Netherlands. Of note, the ‘sawtooth effect’ for both the expected and observed number of cancer diagnoses between early–mid-April 2020 and early June 2020 can be explained, in part, by four official national holidays spanning that period. On these holidays, a broad range of non-essential services, such as routine diagnostic practices, are closed

The number of colorectal cancer diagnoses among individuals aged < 55 or > 75 years (i.e. those not invited for biennial faecal immunochemical testing) was significantly lower than the expected numbers in the first weeks of April (Fig. 1c). Thereafter, it reached and remained at the expected level. In contrast, the number of colorectal cancer diagnoses among individuals aged 55–75 years (i.e. those invited for biennial faecal immunochemical testing) remained slightly lower than the expected number of diagnoses as of early May—that is, 6 weeks after the halt of colorectal cancer screening (Fig. 1d). The observed number of diagnoses ultimately reached the level of the expected values since late June.

The information gleaned by the NCR provides clues that—on top of changes in health-seeking behaviour and referral practices [1]—the temporary halt of national population screening programmes exacerbated fewer breast and colorectal cancer diagnoses in age groups eligible for cancer screening programmes. We cannot yet establish whether diagnostic delays due to the COVID-19 crisis resulted in stage migration. This issue provoked passionate debates—based on the best available literature—about the magnitude of neoplastic progression and cancer deaths amid the COVID-19 pandemic, especially in the light of the extent of the delay [2–5]. The decreased diagnostic scrutiny of cancer amid the COVID-19 pandemic might support resolving controversies regarding overdiagnosis of particular early-stage cancers that would not otherwise become clinically apparent. To address the concerns surrounding the collateral damage of COVID-19 on oncological care in the Netherlands in more detail, information on a variety of patient, tumour, treatment, and survival characteristics will be garnered in the NCR. These data can be compared with data from previous years to assess whether temporal changes in stage distribution and first-line treatment occurred during the COVID-19 crisis.

## Supplementary information

**Additional file 1:** Methological details and additional results.

## Data Availability

The data that support the findings of this study are available via The Netherlands Comprehensive Cancer Organisation. These data are not publicly available, and restrictions apply to the availability of the data used for the current study. However, these data are available upon reasonable request and with permission of The Netherlands Comprehensive Cancer Organisation.

## Abbreviations

COVID-19: Coronavirus disease 2019
PPE: Personal protective equipment
NCR: Netherlands Cancer Registry
DCIS: Ductal carcinoma in situ
