# Supplementary material for: The impact of the temporary suspension of national cancer screening programmes due to the COVID-19 epidemic on the diagnosis of breast and colorectal cancer in the Netherlands
Source: J Hematol Oncol. 2020 Nov 4;13:147. doi: 10.1186/s13045-020-00984-1 (PMC7609826; doi:10.1186/s13045-020-00984-1)

ONLINE SUPPLEMENTARY APPENDIX

Title

The impact of the temporary suspension of national cancer screening programmes due to the COVID-19 epidemic on the diagnosis of breast and colorectal cancer in the Netherlands

Authors and affiliations

Avinash G. Dinmohamed,[1-3] Matteo Cellamare,[1] Otto Visser,[4] Linda de Munck[1] Marloes A.G. Elferink[1] Pieter J. Westenend,[5,6] Jelle Wesseling,[7,8] Mireille J.M. Broeders,[9,10] Ernst J. Kuipers,[11] Matthias A.W. Merkx,[12,13] Iris D. Nagtegaal,[14,15] Sabine Siesling,[1,16]

[1]Department of Research and Development, Netherlands Comprehensive Cancer Organisation (IKNL), Utrecht, The Netherlands, [2]Erasmus MC, University Medical Center Rotterdam, Department of Public Health, Rotterdam, The Netherlands; [3]Amsterdam UMC, Department of Hematology, Cancer Center Amsterdam, Amsterdam, The Netherlands; [4]Department of Registration, Netherlands Comprehensive Cancer Organisation (IKNL), Utrecht, The Netherlands; [5]Department of Pathology, Albert Schweitzer Hospital, Dordrecht, The Netherlands; [6]Laboratory for Pathology Dordrecht, Dordrecht, The Netherlands; [7]Divisions of Diagnostic Oncology and Molecular Pathology, The Netherlands Cancer Institute, Amsterdam, The Netherlands; [8]Department of Pathology, Leiden University Medical Center, Leiden, The Netherlands; [9]Radboud Institute for Health Sciences, Radboud University Medical Center, Nijmegen, The Netherlands; [10]Dutch Expert Centre for Screening, Nijmegen, The Netherlands; [11]Erasmus MC, University Medical Center Rotterdam, Rotterdam, The Netherlands; [12]Board of Directors, Netherlands Comprehensive Cancer Organisation (IKNL), Utrecht, The Netherlands; [13]Department of Oral and Maxillofacial Surgery, Radboud University Medical Center, Nijmegen, The Netherlands; [14]PALGA Foundation, Houten, The Netherlands; [15]Department of Pathology, Radboud University Medical Center, Nijmegen, The Netherlands; [16]Department of Health Technology and Services Research, Technical Medical Centre, University of Twente, Enschede, The Netherlands

Supplementary methods

Statistical analyses

The expected numbers of diagnoses between January 6, 2020, and October 4, 2020, were modelled using a dynamic harmonic regression model (see equation).

$$y_t = \beta_0 + \beta_1 x_{1,t} + \sum_{j=1}^{K} \left[a_j sin\left(\frac{2\pi jt}{52}\right) + b_j cos\left(\frac{2\pi jt}{52}\right) \right] + \eta_t$$

The K Fourier terms are used to model the seasonality, while the short-term time-series dynamics are handled by the ARIMA(p,d,q) error η_t. The number of K Fourier terms and the order of the ARIMA model have been selected by minimizing the corrected Akaike's Information Criterion (AICc). Finally, we account for the effect of public holidays by introducing a discrete covariate $x_{1,t}$ that specifies the number of working days in each week. We aimed to evaluate the effect of the temporary suspension of the national screening programmes on the initial pathological cancer notification of breast cancer—including its pre-cancerous form; that is, ductal carcinoma *in situ*—and colorectal cancer during the COVID-19 epidemic in the Netherlands. In view of this, we compare the observed number of cases during the study period with the expected number given by the estimated model using data from previous years (that is, the period 2010-2019). The analysis has been performed separately for the population eligible for screening and the population not eligible.

For breast cancer, a dynamic regression with 11 Fourier components and ARIMA(1,1,1) error was fitted for the population eligible for biennial mammography screening (that is, women aged 50-74 years), while an ARIMA(3,0,0) with 9 Fourier terms for women not invited for biennial mammography screening (that is, those aged <50 or >74 years). As for colorectal cancer, we fitted a regression with 15 Fourier components and ARIMA(1,1,1)

error among the population invited for biennial faecal immunochemical testing (that is, those age 55-75 years) and ARIMA(1,1,2) error term with 5 Fourier components for those not invited for biennial faecal immunochemical testing (that is, those aged <55 or >75 years).

The validity of the constructed models to forecast the expected values accurately was assessed by juxtaposing the observed number of diagnoses in 2019 with the expected number of diagnoses in 2019, of which the latter is based on data from the period 2010-2018. The results of this validation are graphically presented in Supplemental Figure 1. In brief, the constructed models could predict the expected number of diagnoses in 2019 with high accuracy. Of note, the trends in cancer diagnoses in the period 2019 show an increase and decrease in particular weeks. This phenomenon primarily relates to reduced diagnostic scrutiny during public holidays and summer holidays, and around the turn of the year. The model for the expected number of weekly diagnoses accounts for the increases and decreases objectified in earlier periods.

All statistical analyses were performed by the R statistical software (version 3.6.3; R Foundation for Statistical Computing).

Supplementary figure legend

Supplemental Figure 1. The weekly number of breast and colorectal cancer diagnoses in the Netherlands during the calendar year 2019. The difference between the observed (pink line) and expected number of cancer diagnoses (blue line) is considered statistically significant when the observed number of cancer diagnoses does not fall within the range of the 95% confidence intervals of the expected number of cancer diagnoses (blue shaded area). Panels A and B show the observed and expected number of breast cancer diagnoses among women aged <50 or >74 years (ie, those not invited for biennial mammography screening) and women aged 50-74 years (ie, those invited for biennial mammography screening), respectively. Panels C and D show the observed and expected number of colorectal cancer diagnoses among individuals aged <55 or >75 years (ie, those not invited for biennial faecal immunochemical testing) and individuals aged 55-75 years (ie, those invited for biennial faecal immunochemical testing), respectively. The current statistics do not include cases diagnosed in one of the 74 hospitals in the Netherlands.

Supplemental Figure 2. The weekly number of invasive breast cancer and ductal carcinoma *in situ* diagnoses in the Netherlands between January 6, 2020, and October 4, 2020. The difference between the observed (pink line) and expected number of cancer diagnoses (blue line) is considered statistically significant when the observed number of cancer diagnoses does not fall within the range of the 95% confidence intervals of the expected number of cancer diagnoses (blue shaded area). Panels A and B show the observed and expected number of invasive breast cancer diagnoses among women age <50 or >74 years (ie, those not invited for biennial mammography screening) and women aged 50-74 years (ie, those invited for biennial mammography screening), respectively. Panels C and D show the observed and expected number of *ductal carcinoma in situ* diagnoses among women age <50 or >74 years (ie, those not invited for biennial mammography screening)

and women aged 50-74 years (ie, those invited for biennial mammography screening), respectively. The current statistics do not include cases diagnosed in one of the 74 hospitals in the Netherlands.

Supplemental Figure 1

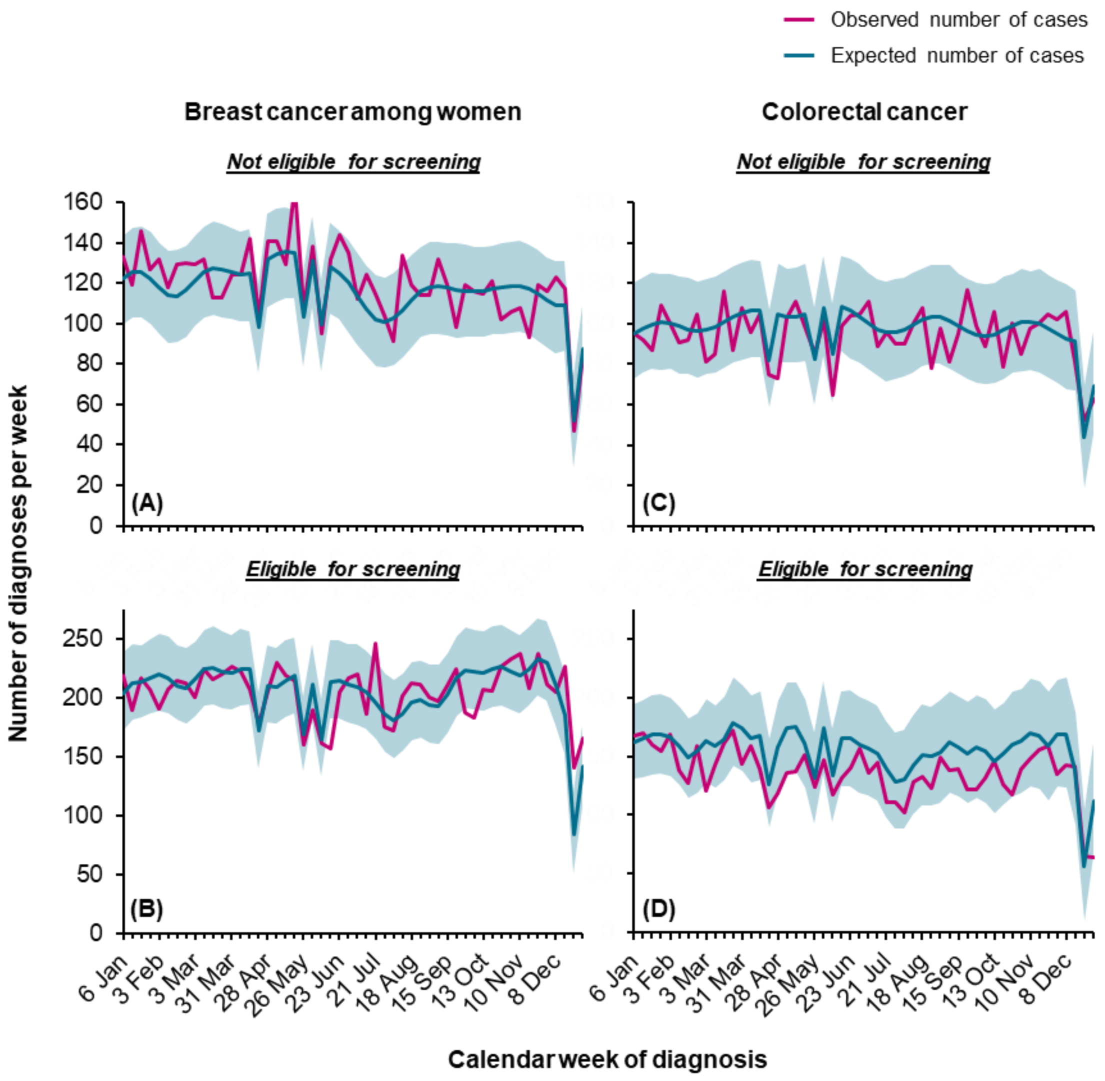

Supplemental Figure 2

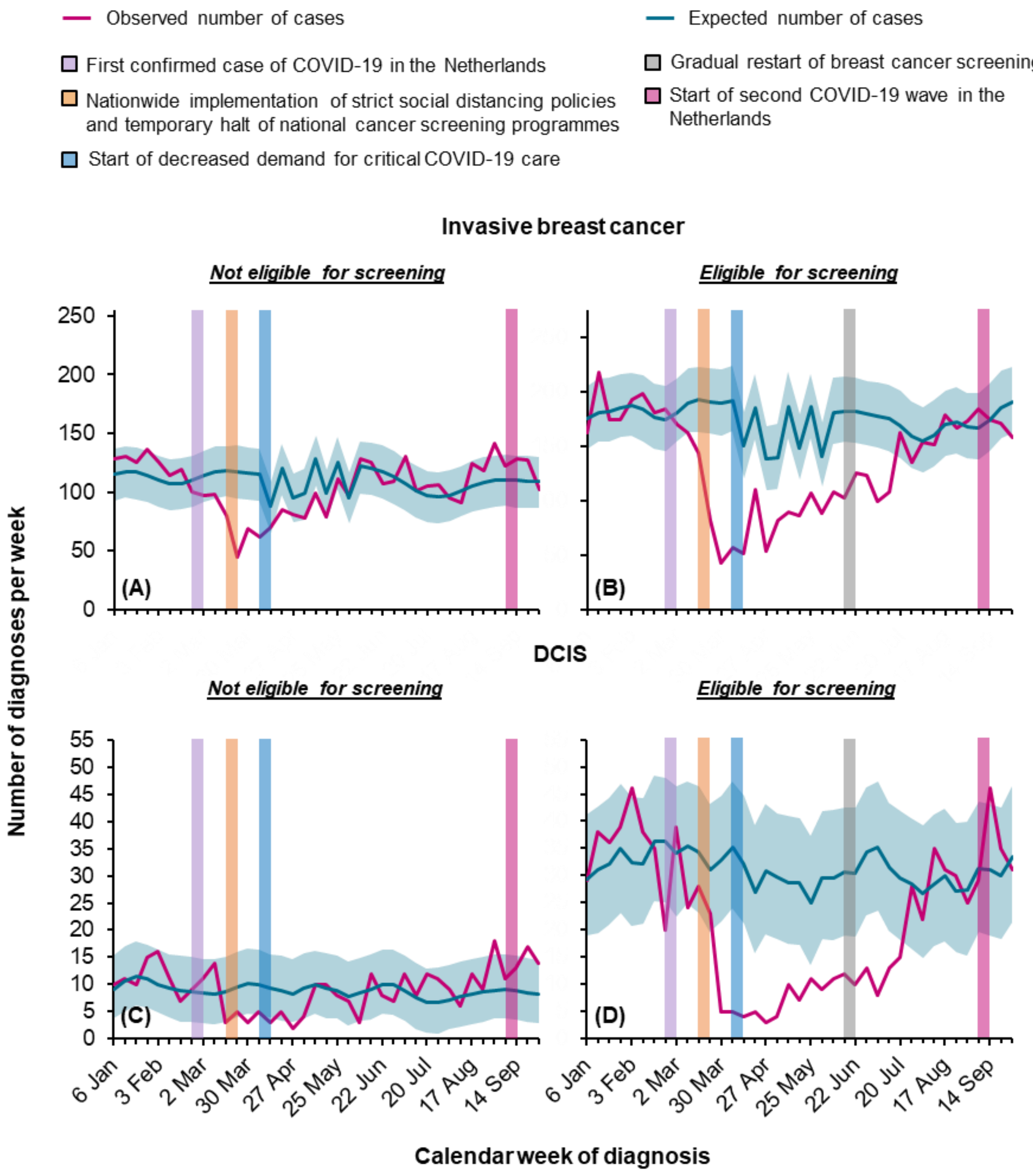

Supplement: Supplementary file 1 — Additional file 1: Methological details and additional results. [file 13045_2020_984_MOESM1_ESM.pdf]
